# Retraction Note: Intravenous lidocaine improves postoperative cognition in patients undergoing laparoscopic colorectal surgery: a randomized, double-blind, controlled study

**DOI:** 10.1186/s12871-024-02835-9

**Published:** 2024-11-28

**Authors:** Xian-xue Wang, Jing Dai, Qi Wang, Hui-wei Deng, Yun Liu, Gui-fan He, Hua-jing Guo, Ya-lan Li

**Affiliations:** 1 Department of Anaesthesiology, The First Affiliated Hospital of Jinan University, Guangzhou, 510632 China; 2 Department of Anaesthesiology, The First People’s Hospital of Changde City, Changde, 415000 China; 3 Hengyang Medical College, University of South China, Hengyang, 421001 China

**Retraction Note: BMC Anesthesiology (2023) 23:243**

10.1186/s12871-023-02210-0

The Editor has retracted this article. After publication, concerns were raised regarding the reporting of this randomised controlled study. An investigation has found that the primary outcome stated in the trial registration record is different from that reported in this article. In addition, there is a difference in the inclusion criteria. Therefore, the Editor no longer has confidence in the results and conclusions presented.

Authors Xian-xue Wang and Ya-lan Li do not agree with this retraction. Authors Jing Dai, Qi Wang, Hui-wei Deng, Yun Liu, Gui-fan He, and Hua-jing Guo have not responded to correspondence about this retraction.

